# Abundance and prevalence of ESBL coding genes in patients undergoing first line eradication therapy for *Helicobacter pylori*

**DOI:** 10.1371/journal.pone.0289879

**Published:** 2023-08-10

**Authors:** Dita Gudra, Ivars Silamikelis, Janis Pjalkovskis, Ilva Danenberga, Darta Pupola, Girts Skenders, Maija Ustinova, Kaspars Megnis, Marcis Leja, Reinis Vangravs, Davids Fridmanis

**Affiliations:** 1 Latvian Biomedical Research and Study Centre, Riga, Latvia; 2 Institute of Clinical and Preventive Medicine, University of Latvia, Riga, Latvia; 3 Faculty of Medicine, University of Latvia, Riga, Latvia; Nitte University, INDIA

## Abstract

The spread of extended-spectrum beta-lactamases (ESBLs) in nosocomial and community-acquired enterobacteria is an important challenge for clinicians due to the limited therapeutic options for infections that are caused by these organisms. Here, we developed a panel of ESBL coding genes, evaluated the abundance and prevalence of ESBL encoding genes in patients undergoing *H*. *pylori* eradication therapy, and summarized the effects of eradication therapy on functional profiles of the gut microbiome. To assess the repertoire of known beta lactamase (BL) genes, they were divided into clusters according to their evolutionary relation. Primers were designed for amplification of cluster marker regions, and the efficiency of this amplification panel was assessed in 120 fecal samples acquired from 60 patients undergoing *H*. *pylori* eradication therapy. In addition, fecal samples from an additional 30 patients were used to validate the detection efficiency of the developed ESBL panel. The presence for majority of targeted clusters was confirmed by NGS of amplification products. Metagenomic sequencing revealed that the abundance of ESBL genes within the pool of microorganisms was very low. The global relative abundances of the ESBL-coding gene clusters did not differ significantly among treatment states. However, at the level of each cluster, classical ESBL producers such as *Klebsiella* sp. for *bla*_OXY_ (*p* = 0.0076), *Acinetobacter* sp. for *bla*_ADC_ (*p* = 0.02297) and others, differed significantly with a tendency to decrease compared to the pre- and post-eradication states. Only 13 clusters were common across all three datasets, suggesting a patient-specific distribution profile of ESBL-coding genes. The number of AMR genes detected in the post-eradication state was higher than that in the pre-eradication state, which could be attributed, at least in part, to the therapy. This study demonstrated that the ESBL screening panel was effective in targeting ESBL-coding gene clusters from bacterial DNA and that minor differences exist in the abundance and prevalence of ESBL-coding gene levels before and after eradication therapy.

## 1. Introduction

Appropriate and inappropriate use of antimicrobials is a well-recognized driver of resistance [1–3] as it can favor the selection of resistant bacteria [4, 5] opening up an ecological niche in which resistant pathogens can flourish [6]. To prevent the spread of resistance and maintain the effectiveness of antibiotics, a number of strategies have been put forth. These included controlled antibiotic use in agriculture, development of disease prevention strategies, improved antibiotic use strategies, development of novel antimicrobials, and others [6, 7]. Despite these measures, new technologies and improved diagnostics are needed to ensure that antimicrobials are used only when necessary to prevent the spread of antibiotic resistance.

Beta-lactam antimicrobial agents, which contain a β-lactam ring in their molecular structure, are the most common treatment option for bacterial infections. However, bacteria that produce beta-lactamase (BL) enzymes can deactivate these antibiotic agents by hydrolyzing the amide bond in the β-lactam ring [8], thus compromising the efficacy of empiric treatment. This deactivation mechanism is employed by a variety of Gram-negative bacteria such as *Escherichia*, *Klebsiella*, *Pseudomonas*, *Acinetobacter*, *Citrobacter*, *Proteus*, and others [9–12]. Continuous exposure of bacterial strains to a multitude of beta-lactam antimicrobials has evoked dynamic and continuous production and mutations of BL in these bacteria, thereby expanding their activity [13]. These enzymes, referred to as extended-spectrum BLs (ESBL), confer multi-drug resistance to a wide range of beta-lactam antibiotics, including penicillin, amoxicillin, cephalosporin, and others [11, 14]. Notably, BL and ESBL genes are often carried by highly mobile plasmids or other types of mobile genetic elements, which can enable their clonal spread among other bacterial species, while retaining resistance genes from other species, thus further limiting treatment options for infections caused by ESBL-producing bacteria [8, 11]. Even common infections, such as infections of the urinary tract caused by ESBL-producing bacteria, necessitate more elaborate treatment. Patients with these infections may require hospitalization and intravenous administration of carbapenem antibiotics, which are typically used as a last resort [15]. Carbapenems have been identified as one of the few remaining antibiotics capable of treating ESBL infections. However, the prevalence of resistance enzymes capable of deactivating these antibiotics has been observed to increase as well [15–17]. Consequently, infections caused by ESBL producers are associated with poor outcomes [18] and increased mortality [19, 20].

ESBLs are widely distributed worldwide, and more than 1.5 billion people are estimated to be colonized with ESBL-producing bacteria [21, 22]. This has led to ESBL-producing bacteria being considered as one of the most pressing public health threats in terms of antimicrobial resistance [15]. The highest prevalence of ESBLs has been observed in the Western Pacific, Eastern Mediterranean, and Southeast Asian regions, while the Americas and Europe display the lowest rates [21, 23]. While developing countries have the highest burden of ESBL-producing bacteria, developed countries are also experiencing an increase in prevalence [22]. Accumulated evidence suggests that risk factors for ESBL colonization include recent antibiotic exposure [24–28], repeated urinary tract infections [26, 27] and use of urinary catheter [26, 27, 29], use of intubation tube [28], recent hospitalization with an emphasis on ICU [27, 30], history of surgical procedures [27, 28, 30, 31], previous colonization with an ESBL-producing bacteria [27, 30], and even drinking water, food, and interaction with domestic animals [32]. Furthermore, antimicrobial use has been reported to be an additional risk factor for ESBL-producing bacterial infections among international travelers [1, 24, 33–36]. This raise concerns in regard to the global spread of ESBLs, thus emphasizing the importance of understanding the distribution and dynamics of the various ESBL encoding genes in order to develop effective control measures and prevent transmission, as well as to decolonize carriers.

The prevalence of *Helicobacter pylori* infection in developing countries is as high as 70% [37], although the rate of infection varies regionally. *H*. *pylori* is a chronic gastric pathogen that frequently colonizes mucosal layers and causes dyspeptic symptoms of varying severity [38]. The current first-line treatment for *H*. *pylori* infection consists of a combination of proton pump inhibitor (PPI) and two antibiotics, among which are amoxicillin, clarithromycin, or metronidazole. They are consumed daily for 7–14 days [38, 39]. Within the eradication scheme, PPIs are used to increase intragastric pH to maintain *H*. *pylori* in a replicative vegetative phase [40], whereas the macrolide antimicrobial agent clarithromycin is used to inhibit bacterial protein synthesis [41] and the beta-lactam antimicrobial agent amoxicillin is used to inhibit bacterial cell wall biosynthesis [42]. In addition, because amoxicillin is a broad-spectrum antibiotic used to treat upper and lower respiratory tract, skin, and other infections [43], its consumption is rapidly increasing worldwide [44].

To date, numerous studies have focused exclusively on the prevalence of ESBL genes in clinical samples of major ESBL producers, such as *Escherichia coli*, *Klebsiella pneumoniae*, *Klebsiella oxytoca* and others. In order to gain further insight into this issue, in the present study we developed a screening panel that targets ESBL genes and employed both targeted and shotgun sequencing methodologies to investigate the abundance and prevalence of ESBL-coding genes in samples from patients undergoing first-line triple antibacterial eradication therapy for *H*. *pylori*. Additionally, the resistome profile of all study participants was evaluated before and after *H*. *pylori* eradication therapy. This is the first study that introduces an ESBL screening panel that is capable of identifying a wide range of ESBL coding genes originating from various microbial groups.

## 2. Methods

### 2.1 Patients, sample collection and storage

**Fig 1:**
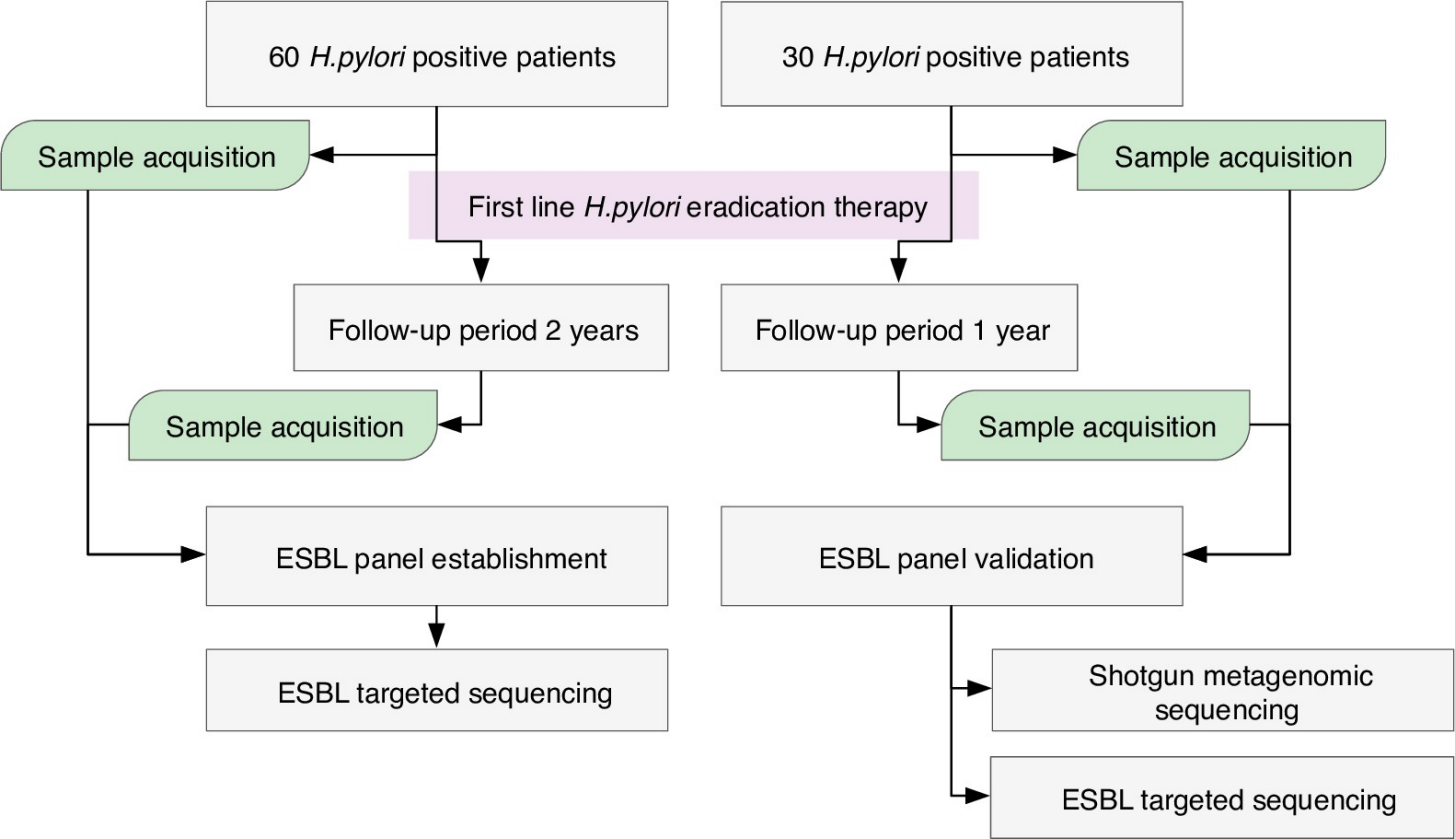
Study design. First line *H*. *pylori* eradication therapy consisted of *esomeprazolum* 40 mg, *clarithromycinum* 500 mg and *amoxicillinum* 1000 mg, each twice per day for ten days.

In total, 90 individuals with positive *H*. *pylori* infection who met the following criteria were included in this study: men and women aged 40 to 64 years; self-reported alcohol consumption 2–3 times a month or less; no history of colon or rectum polyps since age 20, gallstones, gastric cancer, gastric resection, alarm symptoms for digestive or any other diseases, type 2 diabetes, ulcerative colitis, Crohn’s disease, coeliac disease, biliary cirrhosis, thyroid diseases, hepatitis B viral infection or serious psychiatric disorders. A ^13^C-Urea breath test (Euroisotop, Germany) was performed to diagnose *H*. *pylori* infection. A detailed questionnaire was collected from each study participant, which contained information such as age, BMI, medical history (e.g., gastrointestinal diseases, viral infections, autoimmune diseases, and cancer), and lifestyle habits. Samples from 60 individuals collected over two years were used for the ESBL panel experimental group to explore long term variation in the abundance of ESBL coding genes. Samples from the remaining 30 individuals collected over one year were used for validation of the ESBL panel to account for the abundance of ESBL-encoding genes over a shorter time period (Fig 1).

Fecal samples were acquired from each recruited patient before starting the standard first-line *H*. *pylori* eradication (HPE) therapy and one–two years after. Each patient was prescribed the following medications twice a day for ten days: *Esomeprazolum* 40 mg, *clarithromycinum* 500 mg, and *amoxicillinum* 1000 mg. All procedures conformed to institutional ethical standards, and written consent was obtained from all patients prior to enrollment in the study.

In total, 180 samples were obtained within 30 minutes of defecation. Samples were transferred to an OC-Sensor tube (approx. 0.06–0.1 g) (Eiken Chemical Co, Japan), immediately homogenized, and stored at -80°C until further processing according to previously validated storage conditions [45].

### 2.2 DNA extraction

Fecal samples from the OC-Sensor tubes were extracted using a disposable syringe and transferred to pre-labelled 5 mL tubes. Excess OC-Sensor tube solvent was removed from the samples by lyophilization for approximately 15 h in a Christ Alpha 1–2 LD Freeze Dryer (SciQuip Ltd., UK). DNA from the dry remnants of OC-Sensor samples was isolated using the FastDNA SPIN Kit for Soil (MP Biomedicals, USA) according to the manufacturer’s guidelines (S1 File).

### 2.3 ESBL gene cluster primer design

Known BL nucleotide sequences were obtained from the NCBI GenBank (accession date: 02.01.2018). Primer design was performed separately on TEM BLs, as they formed a homogenous group that was highly dissimilar from the rest of the BL sequences. Multiple alignments of the BL sequences were obtained using MAFFT v.7.392 [46]. The number of pairwise non-gapped mismatches between the BL sequences was calculated, and subsequent hierarchical clustering with complete linkage was performed on the distance matrix calculated from the alignment, using the number of non-gapped mismatches divided by the length of the alignment as the distance metric. Clusters were defined at a distance cut-off of 0.1. For each cluster, we identified contiguous conserved regions in the alignment using the Shannon index, for which at most 10% of the nucleotides would differ in each position. Regions with length 18 bases or longer were used in further experiments.

For each cluster, all possible pairs of conserved regions were computed such that the interval between regions in a pair was not longer than 500 bp. Each conserved region pair was scored by summing their Shannon indices at each position and sorted by their scores in ascending order. Primers were then designed on these region pairs with PRIMER3 v.2.4.0, by specifying PCR product sizes in the 200–500 bp range [47]. The best primer pairs for each cluster were evaluated by identifying the potential binding sites in the original list of BL sequences. A site was regarded as a potential binding site if there were up to three mismatches between the primer sequence and template. Additionally, no mismatches with the template were allowed for the last five nucleotides at the 3’ end of the primer. The binding site algorithm was implemented using the SeqAn library [48]. Regions bound by the primers were then aligned against the BL sequence database to evaluate potential off-target PCR products, that is, sequences that mapped against multiple BL clusters. Primer pairs that were overlapping (forming heterodimers estimated with primer3-py) or forming PCR products shorter than 50 bp were identified and pooled into separate sequencing batches. The designed primers were synthesized by MetaBion (Metabion International AG Ltd., Germany).

### 2.4 Sample preparation and targeted sequencing

Pools of primers with equal molarities and volumes were prepared for targeting the ESBL-coding genes and primers for the normalization of ESBL counts- primer pair Probio_Uni-F/Probio_Uni-R targeting *16S rRNA* gene V3 region [49]. PCR amplification of ESBL coding gene regions was performed using a 10 μM custom designed primer pool (S1 Table), Phusion U Multiplex PCR Master Mix (Thermo Fisher Scientific, USA), and GeneAmp® PCR System 9700 (Thermo Fisher Scientific, USA). The reaction mixture was prepared according to the manufacturer’s recommendations, and the thermal conditions were set as follows: 98°C for 30 s; 35 cycles of 98°C for 10 s, 55°C for 30 s, 72°C for 15 s; with a final extension at 72°C for 7 m. The success of the reaction was then assessed by 1.2% agarose gel electrophoresis. 100 ng of the acquired amplicons were used for library generation using the Ion Plus Fragment Library Kit (Thermo Fisher Scientific, USA) and the NucleoMag® NGS Clean-Up and Size Select kit (Macherey-Nagel, Germany) purification module. The quality and quantity of the amplicons were assessed using an Agilent High Sensitivity DNA kit on an Agilent 2100 BioAnalyzer (Agilent Technologies, USA).

Prior to emulsion PCR, each library was diluted to 12 pM and pooled for up to 18 libraries per sequencing run. The Ion PGM^TM^ Hi-Q^TM^ View OT2 kit (Life Technologies, USA) and Ion OneTouch DL instrument (Life Technologies, USA) were used for template generation. Sequencing was performed on an Ion 318 v2 chip and Ion Torrent PGM machine using the Ion PGM^TM^ Hi-Q^TM^ View Sequencing kit (Life Technologies, USA). All procedures were performed according to the manufacturer’s instructions, and each run was expected to produce at least 80’000 reads per sample.

### 2.5 Sample preparation and shotgun sequencing

DNA samples for the shotgun metagenome analyses were normalized to an initial library input of 500 ng and sheared using a Covaris S220 Focused-ultrasonicator (Covaris, USA) to reach an average size of fragments 300 bp. Libraries with average insert sizes of 280 bp were prepared using MGIEasy Universal DNA Library Prep Set V1.0, (MGI Tech Co., China) according to the manufacturer’s recommendations. Quality control of the libraries was assessed using the Qubit High Sensitivity dsDNA assay kit on a Qubit 2.0 instrument (Thermo Fisher Scientific, USA) and the Agilent High Sensitivity DNA kit on an Agilent 2100 Bioanalyzer.

Sequencing depth was calculated to achieve at least 20 million reads per sample (paired end, read length 100 bp). Libraries were sequenced using the DNBSEQ-G400 sequencer and a DNBSEQ-G400RS High-Throughput Sequencing Set PE100 (MGI Tech Co., China) according to the standard workflow.

### 2.6 Data analysis of targeted ESBL coding genes

A sequencing platform-specific adapter clipping of the obtained raw reads was performed with Cutadapt v.1.16 [50]. Targeted sequencing data were then aligned against the curated BL sequence database using Bowtie2 v.2.3.5.1, pre-set at very sensitive [51]. Host reads from shotgun metagenomic sequencing data were filtered using Bowtie2 prior to mapping against BL sequences. *16S rRNA* was quantified with SortMeRNA v.2.1 [52] using *16S rRNA* sequences obtained from RNA central v10 [53] with search query ’rna_type: "rRNA" AND TAXONOMY: "9606" AND length: [19 TO 2000000000]’.

To classify sequencing reads into a specific ESBL cluster, we first created a classification scheme as follows: for each ESBL cluster, we identified regions where we expected the PCR product to form. Each putative product was aligned against all BL sequences to evaluate whether the product was specific to a particular cluster. We regarded putative products as specific if they mapped against sequences from only one cluster. Clusters were merged when a putative PCR product was specific to a set of clusters and if such clusters were not discernible. If multiple PCR products were mapped against the same position within a cluster, we established the alignment score threshold as the minimum score from a set of true positive alignments. An annotation table with cluster reference sequence IDs, start and end coordinates of the corresponding primer product regions, and alignment score thresholds were generated. Sequencing reads were assigned to clusters if overlapped with the coordinates in the annotation table and exceeded the alignment score threshold. Putative PCR products were identified using SeqAn, Pandas and Bowtie2. An annotation table and Python script reading sample binary alignment map files with the Pysam package (https://github.com/pysam-developers/pysam) were used to quantify the read count in each cluster for each sample. The read counts of each BL cluster were normalized to the read counts of the *16S rRNA* gene of a particular sample.

### 2.7 Metagenome data analysis

Quality control and quality trimming of the obtained paired-end reads were performed using FastQC and Trimmomatic v0.39 [54] with a quality threshold of 20 and a minimum read length of 36. Quality filtered sequences were then aligned to the human genome reference GRCh37 (hg19, UID:2758) and sequences matching the human genome were removed using Bowtie2 v.2.3.5.1. The taxonomical profile of the metagenomic dataset was assigned using Kraken2 v.2.0.8 [55] and RefSeq database release 98 [56]. *De novo* read assembling into contigs was performed using the IDBA_UD [57] assembler with the *k*-mer length of at least 50. Generated assembly was evaluated using metaQuast [58]. The assembly database and the local alignment of input reads to assembly was performed using Bowtie2. Open reading frame detection and subsequent annotation was performed using PROKKA v.1.14.6 [59] with the manually curated Swiss-Prot UniProtKB [60] database (accessed 08.02.2021.). During the annotation, predictions of rRNA and tRNA, as well as contigs below 250 nt were excluded. Coordinates of predicted protein-coding features (CDS) were used for quantification against the assembly database using HTSeq [61] and the intersection-nonempty resolution mode. According to previous studies [62–64], metagenomic read counts were standardized using the Transcripts Per Million method [65] with an in-house built python script. Subsequently, from annotation files, CRISPR annotation was removed while contig IDs with the respective product information were retained using in-house built sed and awk scripts. Read counts were joined with the filtered annotation by contig ID column for each sample separately. Next, all samples were merged into a single dataset by annotation column using the Pandas [66] library within the Python environment.

Contigs were used to predict the resistome profile of the study subjects using The Resistance Gene Identifier (RGI) v.5.1.1 along with the Comprehensive Antibiotic Resistance Database (CARD) [67] and the DIAMOND [68] alignment tool. Results were gathered for each sample obtained using the heat map function of RGI, by organizing resistance genes based on the resistance mechanism and gene family. Additionally, hierarchical clustering was performed to cluster samples based on their similarity.

### 2.8 Statistical analysis of taxonomical data

Kraken reports were uploaded to the Pavian v.1.0.0 [69] package and taxonomic entries were filtered out if the sum of the assigned sequences for the taxonomic clade across the samples was below 200. Then, SIAMCAT v.1.9.0 [70] was used to evaluate the association of microbial species between pre- and post-eradication states. Briefly, the dataset of taxonomical entities was separated into two groups: pre-eradication (designated as case) and post-eradication (designated as control). The cut-off for the relative abundance of the species was set to 0.001. The association of microbial species between pre-and post-eradication states was determined using the Wilcoxon test at a significance level of *p*<0.05, with the False Discovery Rate multiple hypothesis correction method. In addition, log-transformed normalization was applied to the abundance matrix of microbial species and the Area Under the Receiver Operating Characteristics Curve (AU-ROC) was used as a non-parametric measure of the enrichment. All the acquired measures of association between the pre- and post-eradication groups were visualized in the SIAMCAT association plot.

Furthermore, the dataset was divided into four groups: F-post-erad denoted subjects of the post-eradication group with ineffective HPE; F-pre-erad denoted subjects of pre-eradication group with ineffective HPE; S-post-erad denoted subjects of post-eradication group with successful HPE and S-pre-erad denoted subjects of pre-eradication group with successful HPE. Alpha diversity metrics (Shannon, Chao1 and Observed) were calculated and visualized using Phyloseq v.1.30.0 [71]. Pairwise comparisons of alpha diversity metrics between the treatment states using the Wilcoxon rank sum test and the Holm P-value adjustment method were performed using the Vegan v.2.5–7 package. Non-metric multidimensional scaling was performed using Phyloseq.

### 2.9 Statistical analysis of amplicon data

The relative abundances of the ESBL clusters between the treatment states were compared using the two-tailed paired t-test in the Vegan v.2.5–7 package. The Kruskal-Wallis test was used to assess the significance in the abundance of individual ESBL clusters between the pre- and post-eradication states with the same package. To explore the clusters overlapping between datasets, cluster IDs which appeared at least once in a sample were extracted from all three datasets. A Venn diagram was constructed using the ggVennDiagram [72] v0.1.9 within the R environment.

### 2.10 Statistical analysis of functional data

UniprotKB entry IDs of the summarized annotation dataset were converted into Gene Ontology (GO) IDs using the UniProt online Retrieve/ID mapping tool (https://www.uniprot.org/uploadlists/). The UniProtKB entries which did not match any corresponding GO ID were removed from the dataset. Next, MaAsLin2 v1.8.0 [73] was used to determine the association of the microbiome functional profile with the treatment state. For the MaAsLin2 analysis, the q-value threshold for significance was set to 0.05, the minimum abundance for each GO term was set to 50, the minimum percentage of samples for which a GO term was detected at a minimum abundance was 25%, the random effect for the model was set to the patient ID, and the fixed effect for the model was treatment state. The significance of the association was controlled using the Benjamini-Hochberg multiple testing correction method.

### 2.11 Ethics Approval Statement

This study was approved by the Biomedical Ethics Committee of the Riga East University Hospital Support Foundation, approval No. 13-A/13 from October 3, 2013.

## 3. Results

Of the 180 samples, three failed during the ESBL-targeted amplicon PCR; therefore, these samples with their respective pairs were removed from further analysis. Thus, a total of 174 samples were sequenced (Table 1). Targeted ESBL quantification in samples from the experimental group resulted in acquisition of 56’418’406 Ion Torrent PGM sequence reads (n = 120, in average 454’987±131’587 reads per sample, two year interval between pre- and post-eradication), while the same analysis for samples from the validation group resulted in acquisition of 20’273’116 Ion Torrent PGM sequence reads (n = 54, in average 375’428±178’727 reads per sample, one year interval between pre- and post-eradication) and 1’522’622’154 DNBSEQ-G400 sequence reads (n = 54, in average 28’196’706±3’943’687 sequences per sample, one year interval between pre- and post-eradication).

**Table 1:** Descriptive summary of the patient-specific parameters.

	Experimental ESBL quantification (n = 60, men/women)	Validation group (n = 30, men/women)
**Gender (n, %)**	26 (43.3%) / 34 (56.7%)	10 (33.3%) / 20 (66.6%)
**Average age**	52.48 ± 6.26	52.2 ± 6.97
**Mean Body Mass Index**	28.387 ± 4.69	29.06 ± 4.75
**Positive *H*.*pylori* status as identified by** ^**13**^**C-Urea breath test before eradication**	26 (43.3%) / 34 (56.7%)	10 (33.3%) / 20 (66.6%)
***H*.*pylori* status as identified by** ^**13**^**C-Urea breath test after eradication**	Positive: 2 (3.3%) / 6 (10.0%)	Positive: 1 (3.3%) / 4 (13.3%)
	Negative: 23 (38.3%) / 29 (48.3%)	Negative: 9 (30%) / 16 (53.3%)
**Asthma**	0 / 2 (3.3%)	0 / 2 (6.7%)
**Experienced Duodenitis**	3 (3.3%) / 4 (6.67%)	0 / 0
**Tuberculosis**	1 (1.67%) / 1 (1.67%)	0 / 0
**Hepatitis A**	3 (3.3%) / 4 (6.67%)	0 / 3 (10.0%)

### 3.1 Experimental setting: Targeted ESBL analysis

The ESBL panel was designed to reflect the prevalence and abundance of the most common ESBL types. A total of 245 gene clusters encoding evolutionarily related ESBLs were targeted by designed primer pool, and primers targeting V3 region of the *16S rRNA* gene were added to the pool to normalize the number of ESBL gene cluster counts (cluster names and respective primer sequences are shown in S1 Table). Analysis of the obtained raw sequence data resulted in 1’787 annotated microbial sources containing strain or clinical isolate IDs with respective BL gene groups, classes and gene names (S2 Table, sheet full annotation). Clusters with identical IDs were merged, and the lowest common ancestor was indicated as the microbial source (S2 Table, sheet merged annotation). Thus, from the 245 ESBL clusters targeted, results were obtained for 265 ESBL clusters, of which twenty clusters most likely represented sequence homology with similar BL gene clusters and thus were assigned to multiple clusters. In the pre-eradication subgroup, 89 ESBL clusters, while in the post-eradication subgroup 106 ESBL gene clusters were not detected in any of the study subject’s samples.

Assessing the presence of ESBL gene clusters in each sample in both treatment states, most clusters were absent, while the presence of a particular ESBL cluster in each sample was mostly dispersed. Examples of disperse clusters include *bla*_PER_, *bla*_MSI_, *bla*_CMH_, *bla*_L1_, *bla*_GES_, *bla*_TMB_, *bla*_VIM_, *bla*_E_, *bla*_PDC_, and others. As a result, each sample was represented by 23 different ESBL gene clusters in the pre-eradication study group and 22 different ESBL gene clusters in the post-eradication study group.

**Fig 2:**
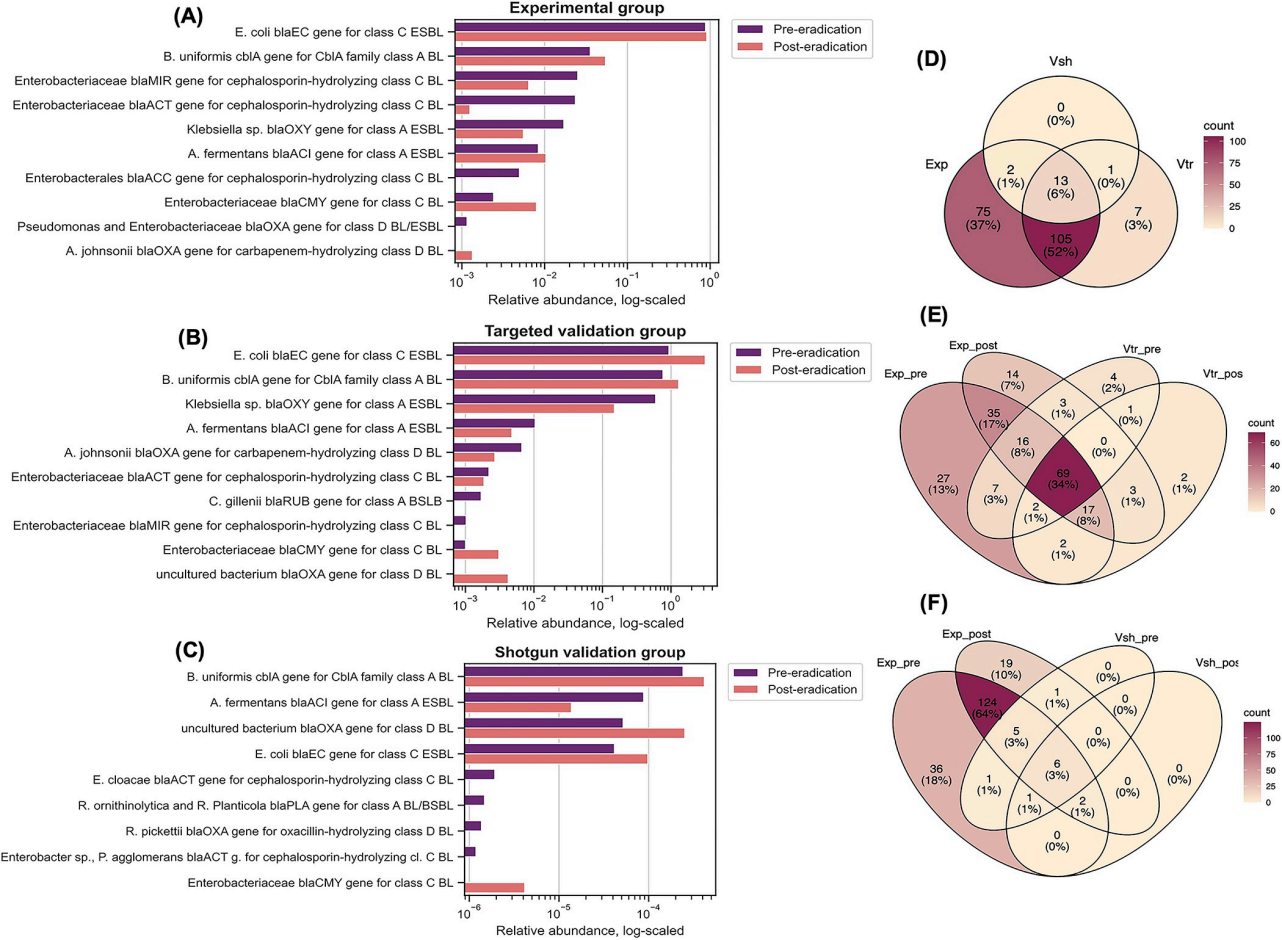
Relative abundance and proportion of common ESBL coding genes between the experimental, validation-targeted, and validation-shotgun groups. Section A-C: relative ESBL coding gene prevalence profile in the pre- and post-eradication samples of the analyzed datasets. For each dataset ESBL counts were normalized against *16S rRNA* gene V3 region bacterial counts and summarized by treatment state. For visualization purposes, for the experimental (A) and targeted validation (B) groups, the abundance threshold was set to 0.1%, and for the shotgun validation (C) group it was 0.0001%. Section D-F: Venn diagram of the ESBL gene cluster counts observed in the experimental group and the validation group of shotgun and targeted sequencing. Only gene clusters that appear at least once in the respective sample group were included in the analysis. Abbreviations: ESBL–extended spectrum beta lactamases, BL–beta lactamases, Exp–experimental group, Vtr–targeted validation group, Vsh–shotgun validation group, Exp-pre–pre-eradication sample set of experimental group, Exp-post–post-eradication sample set of experimental group, Vtr-pre–pre-eradication sample set of targeted validation group, Vtr-post–post-eradication sample set of targeted validation group, Vsh-pre–pre-eradication sample set of shotgun validation group, Vsh-post–post-eradication sample set of shotgun validation group.

Comparing pre- and post-eradication subgroups, the most prevalent clusters (Fig 2A) were the *bla*_EC_ gene group for class C ESBL with an annotated source of *Escherichia coli* (pre = 87.98%, post = 91.11%), *cbl*_A_ gene group for class A BL with annotated source of *Bacteroides uniformis* (pre = 3.56%, post = 5.44%), *bla*_MIR_ gene group for class C BL with annotated source of *Enterobacteriaceae* (pre = 2.52%, post = 0.64%), and *bla*_ACT_ gene group for class C BL with an annotated source of *Enterobacteriaceae* (pre = 2.35%, post = 0.12%). The normalized averaged relative abundance of ESBL genes was not significantly abundant between pre- and post-eradication (*p* = 0.5467). Next, we assessed if non-averaged relative abundance of any particular BL cluster differed significantly across treatment states. Thus, we identified eight clusters that were significantly different between pre- and post-eradication subgroups (Table 2, *p*<0.05). Among these dominated the cluster of class A *bla*_OXY_ group ESBL gene with the annotated origin from 12 different *Klebsiella oxytoca* strains, which had a higher relative abundance in the pre-eradication subgroup than in the post-eradication subgroup (*p* = 0.0076). Similarly, abundance of another strain-rich (n = 4) cluster of the B1 subclass *bla*_IMP_ group metallo-BL genes with annotated origins from *Pseudomonas aeruginosa*, *P*. *fluorescens*, *P*. *putida* and *Acinetobacter baumannii* was identified as a significantly different between the treatment states. However, the relative abundance of this one was noticeably lower than that of the previous one. The relative abundance of the B1 subclass *bla*_IMP_ group metallo-BL genes was significantly higher (*p* = 0.0424) in the post-eradication subgroup than in the pre-eradication subgroup.

**Table 2:** Significant distribution of BL genes among annotated bacterial sources from patients comparing the pre- and post-eradication states.

	No.	Annotated taxonomical source	Cluster number	Gene group	Type of beta-lactamase	Embedded beta-lactamase gene	Normalized average relative abundance		*p*-value
							Pre-eradication	Post-eradication	
Experimental set	1.	*Klebsiella* sp.	69	*bla*_OXY_	Class A ESBL	OXY-4-1, OXY-6-2, OXY-6-3, OXY-6-1, OXY-6-4, OXY-5-1, OXY-5-2, OXY-1-4, OXY-1-6, OXY-1-2, OXY-1-1, OXY-1-3	4572.625	1558.578	0.0076
	2.	*Nocardia farcinica*	85	*bla*_FAR_	Class A ESBL	FAR-1	1.203125	1.25	0.00999
	3.	*Acinetobacter* sp.	166	*bla*_ADC_	Class C BL	ADC-83, ADC-84	0.15625	0	0.02297
	4.	*Streptomyces albus*	86	*bla*	Exo family class A BL	-	0.09375	0	0.02297
	5.	Uncultured bacterium	58	*bla*_LRG_	Class A ESBL	LRG-1	0.0234375	0.0625	0.02298
	6.	Uncultured bacterium	150	*bla*_LRA_	Subclass B3 metallo-BL	LRA-17	0.609375	0.28125	0.04219
	7.	*Pseudomonas* sp. and *A*. *baumannii*	269	*bla*_IMP_	Subclass B1 metallo-BL	IMP-44, IMP-41, IMP-11, IMP-21, IMP-16, IMP-22, IMP-58	0.46875	1.4375	0.04239
	8.	Uncultured bacterium	151	*bla*_LRA_	Subclass B3 metallo-BL	LRA-19	0.21875	0.0625	0.04506
Validation set by PGM targeted sequencing	9.	*Achromobacter xylosoxidans*	130	*bla*_OXA_	Class D BL	OXA-114g, OXA-114c, OXA-114f, OXA-114a, OXA-114e, OXA-114b, OXA-114d	0.0002929	0.00003792	0.01174
	10.	*Stenotrophomonas maltophilia*	125	*bla*_L1_	Subclass B3 metallo-BL	-	0.00016589	0.000088825	0.01491
	11.	*Ralstonia pickettii*	135	*bla*_OXA_	Class D BL	OXA-22	0.000017415	0.001294	0.01547
	12.	*Pseudomonas aeruginosa*	97	*bla*_PME_	Class A ESBL	PME-1	0.0001257	0.000056175	0.02113
	13.	*Chromobacterium piscinae*	77	*bla*_CRP_	Class A ESBL	CRP-1	0.0000030419	0.000020348	0.03198
	14.	*Ralstonia mannitolilytica*	136	*bla*_OXA_	Class D BL	OXA-443	0.000030144	0.0011566	0.03428
	15.	*Nocardia farcinica*	85	*bla*_FAR_	Class A ESBL	FAR-1	0.0000016024	0.000018579	0.03942
	16.	*Bacillus clausii*	100	*bla*_BCL_	Class A BL	BCL-1	0.000024638	0	0.03967
	17.	*Pseudomonas* sp., *A*.*baumannii*	269	*bla*_IMP_	Subclass B1 metallo-BL	IMP-44, IMP-41, IMP-11, IMP-21, IMP-16, IMP-22, IMP-58	0.000033418	0	0.03967
	18.	*Serratia marcescens*	6	*bla*_SRT_	Class C BL	SST-1, SRT-1, SRT-2	0	0.000056522	0.03967

### 3.2 ESBL coding gene quantification using the validation group

The efficiency of ESBL panel was validated through targeted and shotgun metagenomic sequencing of DNA from another independent sample group (54 samples from 27 individuals), which also included pre-and post-eradication subgroups to validate the ESBL gene quantification experimental group. To provide results that are comparable to those of experimental group, we used the same ESBL reference database as before. As in the previous group, in the case of targeted sequencing data analysis, we used the number of reads of the V3 region of *16S rRNA* gene to normalize ESBL quantification data between samples, while in the case of the shotgun metagenomic sequencing, the total number of *16S rRNA* sequences were used for normalization.

Overall, sequencing and data analysis results for the validation group by targeted sequencing were similar to those of the experimental group. A total of 254 ESBL clusters were targeted, but data analysis resulted in identification of 265 ESBL clusters, of which twenty showed sequence homology and thus were assigned to multiple clusters. In total, we identified reads from 101 ESBL gene clusters in the pre-eradication subgroup samples and from 95 ESBL gene clusters in the post-eradication subgroup samples. Considering the arithmetic mean of each ESBL gene cluster within the respective eradication subgroup (Fig 2B), there were only seven clusters that exceeded the 0.05% abundance threshold, from which the most abundant ones were the C ESBL class *bla*_*EC*_ group genes (pre = 40.16%; post = 68.3%), the A BL class CblA family *cbl*_*A*_ group genes (pre = 32.86%; post = 27.94%), and the A ESBL class *bla*_*OXY*_ group genes (pre = 25.76%; post = 3.24%). The normalized average relative abundance of ESBL gene groups was not significantly different between pre- and post-eradication subgroups (*p* = 0.32464).

In contrast, analysis of shotgun metagenomic sequencing data from the validation group samples revealed that only 15 ESBL gene clusters were found in all 54 samples taken together. These included *bla*_R1_, *bla*_CMY_, *cbl*_A_, *bla*_ACI_, *bla*_PLA_, *bla*_CKO_, *bla*_EC_, *bla*_SHV_, *bla*_OXY_, three of *bla*_ACT_, and three of *bla*_OXA_. However, their distribution was mostly dispersed among the samples. In the shotgun dataset, each sample was represented by 13 different ESBL gene clusters in the pre-eradication subgroup and by 8 different ESBL gene clusters in the post-eradication subgroup. Considering the arithmetic mean of all identified ESBL gene clusters in the pre- and post-eradication study subgroups (Fig 2C), 11 in the pre-eradication study subgroup and only five in the post-eradication study subgroup exceeded the 0.1% abundance threshold and the most abundant ones were the A BL class CblA family *cbl*_*A*_ group genes (pre = 56.04%, post = 53.0%), A ESBL class *bla*_*ACI*_ group genes (pre = 20.35%, post = 1.75%), D BL class *bla*_*OXA*_ group genes (pre = 12.02%, post = 32.13%), and C ESBL class *bla*_*EC*_ group genes (pre = 9.62%, post = 12.46%). The normalized average relative abundances of ESBL gene groups did not differ significantly between the pre- and post-eradication subgroups (*p* = 0.20919).

Further we assessed if the relative abundance of individual BL gene clusters differed significantly across the treatment states. Thus, within the targeted dataset, we identified 10 BL gene clusters that were significantly different between the pre- and post-eradication subgroups (Table 2, *p*<0.05). These included a cluster of various A ESBL class *bla*_OXA_ group genes with the annotated origin of *Achromobacter xylosoxidans*, a subclass B3 metallo-BL L1 family *bla*_L1_ group gene with the annotated origin of *Stenotrophomonas maltophilia*, and an oxacillin- hydrolyzing D BL OXA-443 class *bla*_OXA_ group gene with the annotated origin of the *Ralstonia picketii* strain PIC-1. However, within the metagenomic dataset, we were unable to identify any specific BL cluster that differed significantly between treatment states and the most likely reason for that was the low number and low abundance of identified ESBL gene clusters (*p*>0.05).

We also quantified the number of ESBL gene clusters that overlapped between the experimental, targeted validation, and shotgun validation groups (Fig 2D–2F). A global comparison, for example, without considering the pre- and post-eradication subgroups, revealed that all three groups contained 13 clusters (Fig 2D) (*cbl*_A_, *bla*_PLA_, *bla*_EC_, *bla*_ACI_, *bla*_OXY_, *bla*_CMY_, *bla*_R1_, three of *bla*_OXA_, and three of *bla*_ACT_). The highest number of shared ESBL gene groups was found between the experimental and targeted validation datasets (Fig 2E). Conversely, the shotgun dataset contained a notably lower amount of ESBL gene groups, resulting in lower coverage compared to the experimental dataset (Fig 2F). Thus, 69 ESBL gene groups were found between the experimental and targeted validation datasets, and only 6 shared ESBL gene groups were found between the experimental and shotgun validation datasets.

### 3.3 Taxonomical characterization

As our analyses included metagenomic sequencing, we also explored the taxonomic and functional composition of patient samples before and after *H*. *pylori* eradication therapy. A detailed taxonomic analysis of samples from the experimental group (n = 120) is described in our previous publication (Gudra et al., 2020). However, to be brief, we found that the dominant genera in the pre-eradication study subgroup were *Bacteroides* (10.3%), *Oribacterium* (9.08%), *Prevotella* (6.16%) and *Parasutterella* (4.87%), whereas in the post-eradication study subgroup–*Bacteroides* (9.75%), *Streptomyces* (7.75%), *Oribacterium* (7.41%) and *Prevotella* (5.81%). Alpha and beta diversities did not differ significantly between the pre-and post-eradication subgroups (*p*>0.05). Moreover, despite receiving eradication therapy, eight of the 60 individuals continued to test positive for *H*.*pylori*, and the available data show that there were no instances of recurrence.

**Fig 3:**
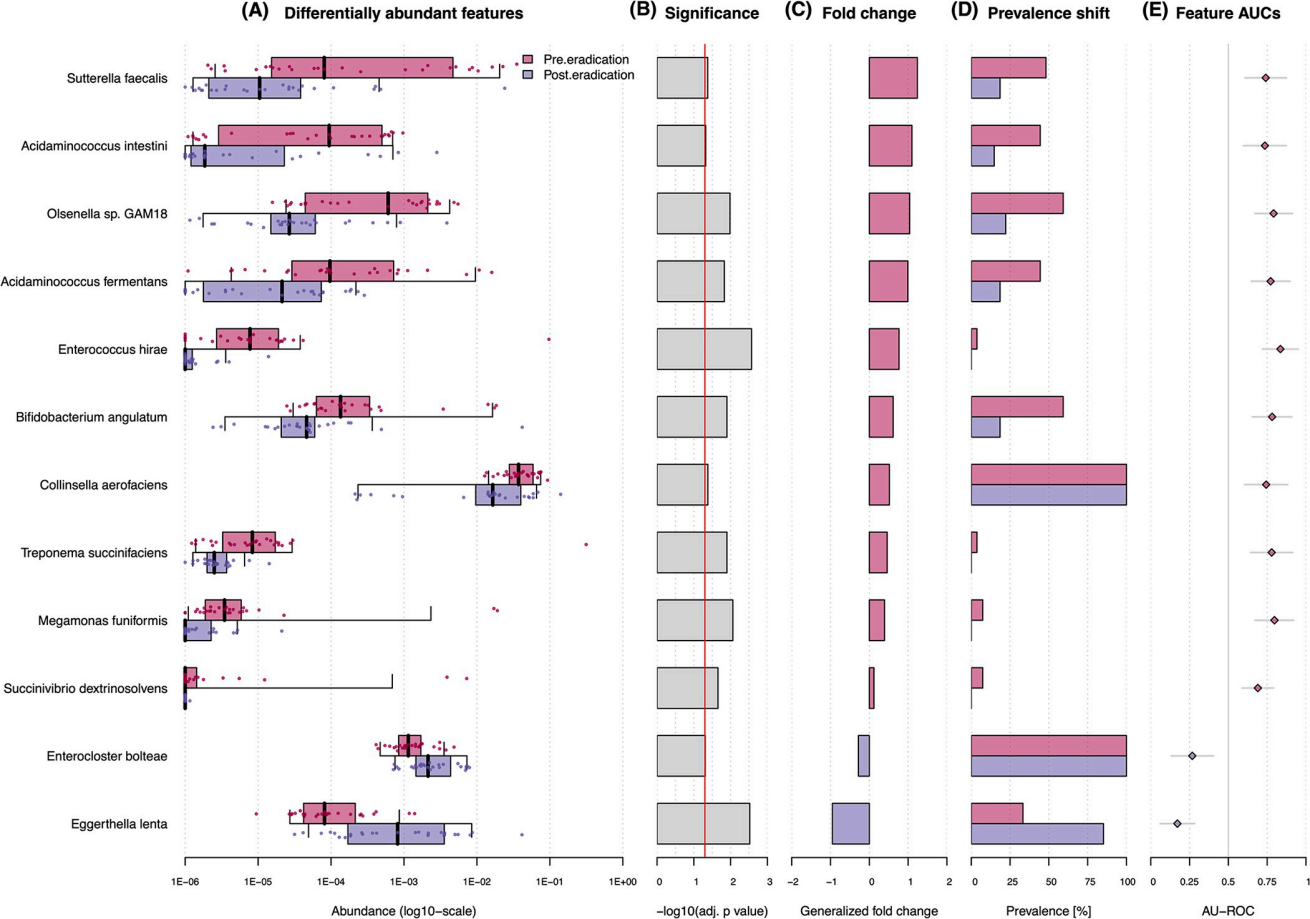
Significantly associated microbial species between the pre- and post-eradication states (at a significance level *p*<0.05). Section A–differentially abundant microbial species between pre- and post-eradication states; Section B–the significance of the enrichment calculated by a Wilcoxon test with FDR multiple hypothesis correction; Section C–generalized fold change of each significantly associated microbial species; Section D–the prevalence shift of each significantly associated microbial species; Section E–the Area Under the Receiver Operating Characteristics Curve (AU-ROC) as a non-parametric measure of the enrichment.

In the current validation group (n = 27), 98.68±1.24% of taxonomic entries belonged to bacteria, 0.33±0.48% to viruses and 0.0026±0.003% to fungi and protozoa combined. A detailed summary of the taxonomy is provided in S1 File, but the most prevalent bacterial genera in the pre-eradication subgroup were *Bacteroides* (37.73%), *Faecalibacterium* (11.86%) and *Bifidobacterium* (6.67%), whereas in the post-eradication subgroup dominated *Bacteroides* (44.95%), *Faecalibacterium* (12.43%), and *Alistipes* (4.73%). We also found that at the species level, the most prevalent bacteria in the pre-eradication subgroup were *Faecalibacterium prausnitzii* (13.45%), *Bacteroides vulgatus* (10.38%), *Bifidobacterium adolescentis* (4.89%) and *Bacteroides uniformis* (4.53%), whereas in the post-eradication subgroup *Faecalibacterium prausnitzii* (14.21%), *Bacteroides vulgatus* (13.35%), *Bacteroides dorei* (6.12%) and *Bacteroides uniformis* (6.11%). Furthermore, we also tested if there was an association between microbial species and treatment state, and according to the acquired results, 12 microbial species were found to be differentially abundant between the pre- and post-eradication subgroups (Fig 3). Of these, two had increased relative abundance (*E*. *bolteae*, *p*_adj._ = 0.049; *E*. *lenta*, *p*_adj._ = 0.003), and 10 had decreased relative abundance (*S*. *faecalis*, *p*_adj._ = 0.042; *A*. *intestini*, *p*_adj._ = 0.048; *Olsenella* sp. GAM18, *p*_adj._ = 0.01; *A*. *fermentans*, *p*_adj._ = 0.015; *E*. *hirae*, *p*_adj._ = 0.003; *B*. *angulatum*, *p*_adj._ = 0.013; *C*. *aerofaciens*, *p*_adj._ = 0.042; *T*. *succinifaciens*, *p*_adj._ = 0.013; *M*. *funiformis*, *p*_adj._ = 0.009; *S*. *dextrinosolvens*, *p*_adj._ = 0.022) in the post-eradication subgroup compared to the pre-eradication subgroup.

Given that the majority of ESBLs detected during this work originated from the *Enterobacteriaceae* family, we decided to compare the abundance of enterobacteria between treatment states in the metagenomic dataset. Our ESBL targeted sequencing data showed that the *bla*_EC_ gene group with an annotated source of *E*. *coli* predominated in both treatment states, but in our metagenomic data, the incidence of *E*. *coli* was only 0.129% before and 0.338% after the eradication therapy. However, although this change in abundance is indeed low, it represents 2.6-fold difference (*p*>0.05). The relative abundance of entire *Enterobacteriaceae* family was 1.29% in the pre-eradication state and 2.44% in the post-eradication state, representing a 1.9-fold difference in favor of the post-eradication state (*p*>0.05). Further on assessing BL genes with significant distribution (Table 2) among the treatment states, *Klebsiella* sp. was one of the dominant annotated sources of ESBLs (Fig 3A, 3B). In metagenomic data, relative abundance of *Klebsiella* sp. was 0.062% in the pre-eradication state samples and 0.022% in the post-eradication state samples, which represents a 2.9-fold difference and agrees well with the targeted sequencing results. Bacterial species such as *Pseudomonas aeruginosa* (pre = 0.005%, post = 0.006%), *Ralstonia picketii* (pre = 0.014%, post = 0.004%) and *Serratia marescens* (pre = 0.0001%, post = 0.0011%) were at very low abundance in all samples, whereas *A*. *xylosoxidans*, *S*. *maltophilia*, *C*. *piscinae*, *R*. *mannitolilytica*, *N*. *farcinica*, *B*. *clausii* and *A*. *baumannii* were not found in the metagenomic dataset. When comparing the outcomes of metagenomic sequencing with targeted sequencing, the observed variance may be due to differences in applied wet-lab and data analysis methodologies, as well as to insufficient sequencing depth to detect very low abundance microorganisms.

Assessing Shannon and Chao1 indices and the observed OTUs (S1 File), only the Shannon index differed significantly between the pre- and post-eradication subgroups (*p*_adj._ = 0.019). Furthering, non-metric multidimensional scaling was applied to assess sample-to-sample variability (S1 File). The analysis revealed non-specific clustering of samples at the species level, suggesting mutual sample similarity irrespective of gender and treatment state. In addition, four of the 27 study subjects continued to test positive for *H*.*pylori* after receiving the eradication medication, and the data that are available indicate that there were no incidences of recurrence.

### 3.4 Functional analysis of the metagenomic profile

**Fig 4:**
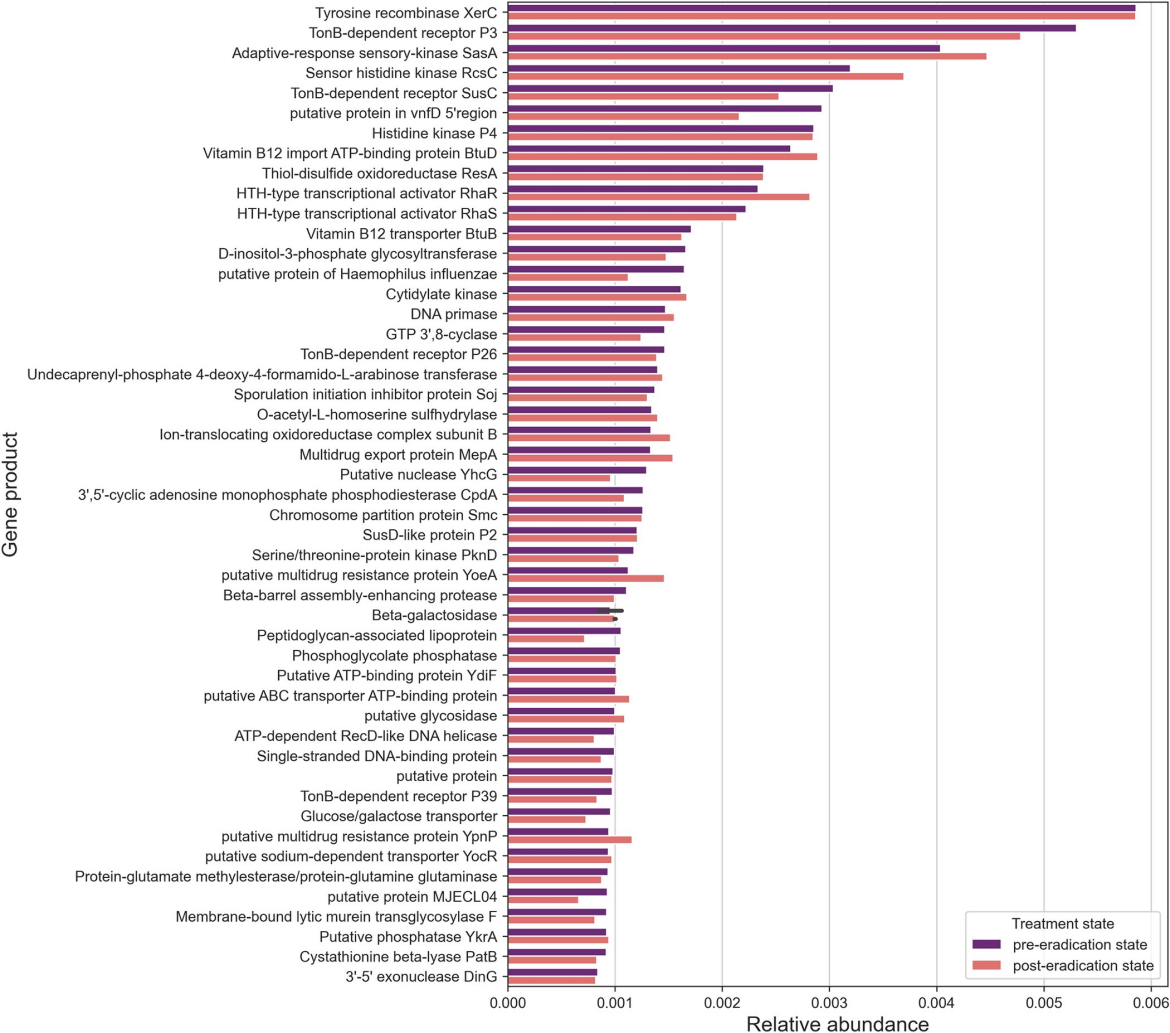
Top 50 most abundant gene products obtained by shotgun metagenomic sequencing between the pre- and post-eradication states.

The functional profiles of the samples were very similar across treatment states, with the predominant gene products being tyrosine recombinase XerC (in both pre- and post-eradication subgroups: 0.59%), TonB-dependent receptor P3 (pre = 0.53%, post = 0.48%), adaptive response sensory kinase SasA (pre = 0.4%, post = 0.45%), the sensor histidine kinase RscC (pre = 0.32, post = 0.37%) and the TonB-dependent receptor SusC (pre = 0.32%, post = 0.25%) (Fig 4). Furthermore, the resulting UniProt IDs were converted to Gene Ontology (GO) annotations, and the original annotations that did not have the corresponding GO IDs were removed from the dataset. Thus, in total 1’993 unique GO IDs were obtained. To assess the relationship between functional profile and treatment state, association analysis was performed revealing 18 GO ID entries that were significantly different between pre- and post-eradication subgroups (S1 File). Significant associations between GO IDs and treatment state were found for entities related to molecule (GO:0035442, *q*-value = 0.012; GO:0034219, *q*-value = 0.012) and ion transport (GO:0006811, *q*-value = 0.005; GO0042777, *q*-value = 0.012; GO:0015693, *q*-value = 0.013; GO:0006817, *q*-value = 0.043), several biosynthetic processes (GO:0019242, *q*-value = 0.032; GO:0045226, *q*-value = 0.043), including cobalamin (GO:0009236, *q*-value = 0.032) and dTMP (GO:0006231, *q*-value = 0.044) biosynthesis, DNA restriction-modification system (GO:0009307, *q*-value = 0.012), metabolic processes (GO:0019568, *q*-value = 0.044; GO:0006541, *q*-value = 0.044; GO:0019243, *q*-value = 0.032), DNA-templated transcription and initiation (GO:0001123, *q*-value = 0.032), ribosomal small subunit biogenesis (GO:0042274, *q*-value = 0.012), adenine salvage (GO:0006168, *q*-value = 0.044) and cellular phosphate ion homeostasis (GO:0030643, *q*-value = 0.044). Notably, most of the abundances of significant GO IDs entities were increased in the post-eradication subgroup (n = 15), while only a few were increased in the pre-eradication subgroup (n = 3). GO entities that exhibited increased abundance in the pre-eradication subgroup and decreased abundance in the post-eradication subgroup were related to ribosomal small subunit biogenesis, DNA-templated transcription and initiation, and adenine salvage.

The diversity of antimicrobial resistance (AMR) genes found in the metagenomic sample set was revealed by additional mapping to the CARD database. Thus, we identified 54 gene families associated with AMR, conferring five different mechanisms of resistance (S3 Table). A total of 1’080 AMR genes were detected in the pre-eradication subgroup, of which 125 had 100% sequence similarity and 955 had strictly significant (≥95% identity) sequence similarity to the CARD reference database. We detected 1’265 AMR genes in the post-eradication subgroup, of which 179 had 100% sequence similarity and 1’086 had strict (≥95% identity) sequence similarity to the CARD reference database. AMR genes present in at least 70% of samples in each treatment state (n = 20) were the antibiotic target alteration genes *ErmB* and *ErmF*; antibiotic inactivation genes chloramphenicol acetyltransferase, *aadS* and *lnuC*; antibiotic efflux genes *Mef(En2)*, *tet(40)*, and *adeF*; antibiotic target alteration gene *rpoB*; antibiotic target protection genes *tet(W/N/W)*, *tet32*, *tetM*, *tetO*, *tetQ*, and *tetW*; and the antibiotic target replacement gene *dfrF*. AMR genes present in all patient samples in the pre-eradication subgroup were antibiotic efflux gene *adeF*, antibiotic target protection gene *tetQ*, and antibiotic target replacement gene *dfrF*. In contrast, AMR genes present in all patient samples of the post-eradication study subgroup were the antibiotic target alteration gene *ErmF*; antibiotic efflux genes *tet(40)* and *adeF*; antibiotic target protection genes *tetO*, *tetQ*, and *tetW*; and the antibiotic target replacement gene *dfrF*. After applying a hierarchical clustering analysis, 12 samples collected from different individuals and within a sample cluster showed a similar reservoir of detected AMR genes. Only two study subjects had pre- and post-eradication subgroup samples, respectively. The identified gene groups included rifamycin-resistant beta-subunit of RNA polymerase (*rpoB*) genes, resistance-nodulation-cell division antibiotic efflux pump genes, *pmr* phosphoethanolamine transferase genes, multidrug and toxic compound extrusion transporter genes, major facilitator superfamily antibiotic efflux pump genes, macrolide phosphotransferase genes, *ampC*-type BL genes, general bacterial porins with reduced permeability to BL genes, ATP-binding cassette antibiotic efflux pump genes, and others.

## 4. Discussion

The misuse of antibiotics has led to a rapid increase in antimicrobial resistance among clinically relevant microorganisms, particularly Gram-negative bacteria [74–78]. Resistance to beta-lactam antibiotics is an emerging problem in healthcare due to extremely limited therapeutic options. Additionally, resistance to this group of antimicrobials is often associated with resistance to other drugs [78, 79]. The emergence of ESBL enzymes highlights the importance of understanding how the associated genes could alter the abundance in gastrointestinal microbiome under prolonged antibiotic pressure. As this phenomenon is difficult to study using culturomics-based approaches and subsequent detection of phenotypes, due to unknown cultivation requirements of most members of the gastrointestinal microbiome, the only alternative is to employ molecular biology approach. Therefore, we developed a panel for detection and long-term abundance and prevalence assessment of BLs in *H*. *pylori-*infected patients before and after a single eradication event.

We were able to identify the majority of targeted ESBL clusters in samples from the experimental cohort. However, there were also some BL gene clusters that were not detected by our panel. Although the most obvious explanation for this would be the absence of a target in the pool of extracted bacterial DNA, it is also possible that, in some cases, the primer bound primarily to the off-target region due to high sequence homology or failed to bind entirely to intended target due to such wet-lab-related aspects as incompatibility with the annealing temperature used. However, detailed examinations involving qPCR or even digital PCR would be necessary to assess the validity of these speculations. Although some studies have used a *16S rRNA* gene copy normalization method to improve the estimation of the actual relative abundance of taxonomic groups, these attempts have so far been unsuccessful because bacterial taxa can have variable numbers of *16S rRNA* gene copies [80, 81]. However, the list of genetic elements in Bacteria that would display sufficient sequence conservation across all taxa is limited, therefore, in this work, *16S rRNA*-based normalization was performed to standardize sequencing results between samples.

The results of this study showed that the majority of BLs originated from Gram-negative bacteria from the genus *Enterobacteriaceae*. This finding is consistent with the results of several studies that demonstrated that the prevalence of ESBL-producing *Enterobacteriaceae* is increasing, even in healthy asymptomatic individuals [82, 83], where it is thought to serve as a reservoir for the spread of ESBLs. The most abundant and prevalent ESBLs in the experimental group were *bla*_EC_ from carbapenem-targeting class C ESBLs [84], the cephalosporinase gene *cbl*_A_ from class A BL targeting ESBLs [85] and *bla*_ACI_ from class A ESBLs [84]. As infections caused by ESBL producers are associated with increased mortality, length of hospital stays and increased treatment costs [86–88], the widespread identification of ESBLs in our patient samples suggests, that it is indeed a growing problem in today’s society. To date, a large proportion of ESBL types have been poorly characterized and the focus is mainly on those of clinical relevance. Therefore, decoding the effects of ESBLs in the host or environment in the context of our study is challenging. However, large-scale identification of ESBL genes in patient samples by the most sensitive molecular methods can still provide valuable information about their epidemiology.

Considering the abundance of ESBL clusters at the level of each sample, we found that most were absent in the individual samples of all three datasets, with the lowest number of clusters found in samples from the metagenomic dataset. While this might seem a striking result, we also observed that there are several overlapping clusters between the datasets, possibly reflecting the core population of ESBLs characteristic for our patient group. Four of these clusters originated from class A, three from class C, one from class D, and one from an unknown BL class. Furthermore, the number of ESBL clusters differed between treatment states. In all three groups (experimental, validation-targeted, and validation-metagenomic)–the number of ESBL gene clusters in the pre-eradication state was higher than in the post-eradication state, suggesting that some ESBL producers could be eliminated during the eradication therapy, resulting in an overall decrease in BL producers. Given that abundant bacterial species (e.g., the core microbiome) are relatively stable over time, statistically significant changes mainly affect bacteria with low abundance. Thus, it is plausible that these ESBL producers belonged to low-prevalence bacteria such as *Enterococcus hirae*, which has been reported to be tolerant to beta-lactam antibiotics [89, 90], and *Megamonas funiformis*, which in multiple cases has been reported as a carrier of various beta-lactamase genes in its genome; for both bacteria, their abundance was significantly reduced in the post-eradication state compared to the pre-eradication state [91]. On the other hand, the abundance of *Enterocloster bolteae* and *Eggerthella lenta* was increased in the post-eradication group. *E*. *bolteae* has been shown to be capable of producing beta-lactamases [92, 93], as was *E*. *lenta* [94]. Generally, monotherapy is used to treat common infections such as pneumonia [95] and urinary tract infections [96], while dual antimicrobial treatment is used for *H*. *pylori*. Therefore, it is plausible that the use of two types of antimicrobial agents may have a synergistic effect that contributes to the reduction of BL producers [97]. If this is true, it is possible that one way to limit the spread of antimicrobial resistance would be to use multiple antimicrobial agents at the same time, not only for *H*. *pylori* infection, but also for other infectious diseases.

In this study, we were able to detect several ESBL clusters whose relative abundances differed significantly between pre- and post-eradication states in both targeted datasets. The annotated taxonomic source indicated that these ESBLs originate from both–Gram-positive and Gram-negative bacteria, such as *Klebsiella* sp., *Pseudomonas* sp., *Acinetobacter* sp., *Achromobacter xylosoxidans*, *Stenotrophomonas maltophilia* and others. Most of these are known ESBL producers, suggesting gastrointestinal carriage and asymptomatic colonization of these organisms. Although the top abundant BL groups were common between the treatment states, the annotated taxonomic source of BL differed between the experimental and targeted validation groups, indicating that resistance genes in the post-eradication group were uptaken from the environment during the reestablishment of gut microbiome. These results were further supported by taxonomic profiles of the metagenomic dataset, which showed that some bacterial species whose ESBL clusters differed significantly between treatment states were increased in the post-eradication state (e.g., *Enterobacteriaceae* family, *Pseudomonas aeruginosa* and *Serratia marescens*), while for some the trend was opposite (e.g., *Escherichia coli*, *Klebsiella* sp. and *Ralstonia picketii*). Considering that these were long-term evaluations and that the mean age of the current patient group was 52 years, and that our previous study [98] uncovered stronger gut microbiome associations with patient-specific characteristics such as age, individual, gender, and medical history, it is plausible that the reservoir of BLs and possibly other AMR genes changes more dynamically than the bacteria themselves between both–Gram-positive and Gram-negative bacteria. Therefore, our findings suggest that in the future greater emphasis should be placed on the development of novel probiotic products and procedures for the controlled gut microbiome reestablishment. These products should ensure that patients’ guts are colonized by as few resistant microorganisms as possible, thus mitigating the situation where our microbiota serves as a reservoir of ESBLs. In addition, healthy lifestyle choices must be added to control the restoration of a non-resistant gut microbiome. Various living conditions, fluctuating lifestyles, diet, physical and/or outdoor activities, mental stress and other factors have been shown to contribute to the health of microbiome and host [99–101], therefore, during the period of microbiome restoration, reinforced attention should also be paid to appropriate lifestyle choices.

Considering the prevalence of ESBL clusters, the highest number of clusters was found in the experimental and targeted validation groups. There were only a few ESBL gene clusters in the metagenomic dataset, suggesting that ESBL abundance is at a very low level. Accurate analysis of the presence or absence of each cluster is possible by amplifying the perspective BLs or by increasing the target read count in metagenomic analyses. However, the amplification step in ESBL panel-based sequencing library preparation may lead to biases in the abundance of some ESBL gene clusters, especially those that share sequence homology.

In this study, we also evaluated the effect of *H*. *pylori* eradication therapy on the taxonomic composition of the gut microbiome using data obtained from shotgun metagenomic sequencing. We found that the microbiome diversity rate was significantly higher in the pre-eradication state than in the post-eradication state. This observation is consistent with literature data, which show that the gut microbiota is significantly altered immediately after eradication therapy and gradually restores to the baseline parameters over time; however, certain alterations may persist up to a year after completion of eradication therapy[98, 102–104]. Although we observed that diversity in the post-eradication state was lower than in the pre-eradication state, we did not observe significant changes in the beta diversity analysis, suggesting that the global composition of microbial communities was highly similar between the pre- and post-eradication states. Indeed, the relative abundance of bacterial species was highly similar between the eradication states and were dominated by *Faecalibacterium prausnitzii*, *Bacteroides vulgatus*, *B*. *dorei* and *B*. *uniformis*. Previous studies have suggested that some *F*. *prausnitzii* genogroups might contain class A BL [105], which was also the dominant BL class in the shotgun validation group, while *Bacteroides* sp. is a well-known group of BL-producing bacteria [106, 107]. In addition, *cbl*_A_ gene of class A BL with an annotated taxonomic source of *B*. *uniformis* was the predominant BL gene in the shotgun validation group, and its abundance was higher in the post-eradication group. In addition, during the association analysis, the abundance of certain microbial species varied between the pre- and post-eradication states. For instance, in the pre-eradication subgroup, we observed increased levels of *Acidaminococcus intestinalis*, previously shown to be elevated in overweight adults [108]. Similarly, we detected increased levels of *Collinsella aerofaciens* and *Treponema succinifaciens* in the pre-eradication subgroup. The former has been associated with low dietary fiber intake and anti-inflammatory effects on the intestinal epithelium [109, 110], while the later was enriched in traditional rural populations [111]. Altogether, minor differences exist in the composition of the microbiome between the pre- and post-eradication states, although these differences may be more related to the diet and general state of health than to the eradication therapy itself. However, in-depth studies involving the reconstruction of bacterial genomes from metagenomic data would be necessary to correlate bacterial abundance with ESBL gene abundance.

In addition to evaluating and validating the ESBL screening panel, we investigated the functional profile and resistome of the microbiome. Thus, we were able to show that the abundance of several genes was increased in the post-eradication state. These genes have a role in the transport of molecules and ions; biosynthetic processes of cobalamin and extracellular polysaccharides; and methylglyoxal, arabinose, and glutamine metabolism. All the above-mentioned processes have a positive and beneficial effect on the human host, most profoundly in the case of cobalamin and extracellular polysaccharides. Additionally, our data also suggests that the abundance of several genes was decreased in the post-eradication state. These genes have a role in the DNA restriction-modification system, DNA-templated transcription and initiation, as well as in the biogenesis of ribosomal small subunits. Their increased levels may be related to the active reproduction of bacteria and the protection of their genome against the invasion of foreign DNA. In addition, since we detected minor differences in the abundance and prevalence of ESBL gene clusters between treatment states, we also evaluated the entire resistome profile. The number of AMR genes detected in the post-eradication subgroup was higher than in the pre-eradication subgroup. Thus, it is apparent that AMR gene diversity has increased under the pressure of antimicrobial therapy. All samples from the pre-eradication subgroup contained three AMR genes–the resistance-nodulation-cell division antibiotic efflux pump gene *adeF*, the tetracycline-resistant ribosomal protection protein gene *tetQ*, and the trimethoprim resistant dihydrofolate reductase dfr gene *dfrF*, while four were detected in all samples from the post-eradication subgroup: Erm 23S rRNA methyltransferase gene *ErmF*, the major facilitator superfamily antibiotic efflux pump gene *tet(40)*, and the tetracycline-resistant ribosomal protection protein genes *tetO* and *tetW*. Furthermore, AMR genes conferring macrolide resistance increased in the post-eradication subgroup. In one study subject, we were able to detect *Chlamydia trachomatis* 23S rRNA with mutations conferring resistance to macrolide antibiotics such as clarithromycin, which was prescribed to study participants in the current study. Although *C*. *trachomatis* is commonly associated with sexually transmitted diseases [112], it has also been shown that the human gastrointestinal tract might be a site of persistent infection with this pathogen [113, 114]. Moreover, in the post-eradication state, we observed an increase in AMR gene families, macrolide esterase and macrolide phosphotransferase, both of which contribute to the inactivation of macrolide antibiotics. This observation might be of increased importance because other studies have shown that previous macrolide use (even 10–12 years ago) correlates with low *H*. *pylori* eradication rates with clarithromycin-based triple antibiotic therapy [115, 116], thus emphasizing the ability of resistance genes to persist in the intestinal tract. In addition, functional analysis confirmed that the relative abundance of gene products involved in the spread of resistance, such as tyrosine recombinase, and gene products involved in signal transduction pathways, such as sensor histidine kinase, was increased in post-eradication state. Given that a sensor histidine kinase can sense the presence of antibiotics and activate transcription of AMR genes, these results, together with elevated levels of potential multidrug resistance proteins, highlight the need for increased attention to AMR gene distribution and dynamics using multi-omics approaches. To date, some studies have reported alteration in the number of AMR genes after *H*. *pylori* eradication therapy [117, 118], however, little is known about the functional mechanisms of gut microbiome dynamics after antibiotic treatment in the long term.

Despite all this wealth of knowledge, this study had several limitations. First, some targeted ESBL-coding gene clusters were absent in all patient samples, and the reason behind their absence remained ambiguous. Therefore, the designed primers that targeted ESBL-coding gene clusters should be further validated using methods such as RT-qPCR or digital PCR. Subsequently, although the validation group was able to mimic the experimental group, greater patient involvement is needed to increase the resolution of the diversity and abundance of genes encoding ESBLs and AMRs, particularly in the metagenomic dataset. In this study, we also observed that some individuals remained *H*. *pylori*-positive after eradication therapy, but the sample size was too small to confirm this observation. Lastly, this study did not assess resistance to the prescribed antibiotics, amoxicillin and clarithromycin, and did not include *H*. *pylori* genomic characterization. However, considering the high prevalence of *H*. *pylori* in the Latvian population, we believe that such studies are of paramount importance and should be addressed in the near future.

Our study suggests that an NGS-based large-scale screening panel of ESBL-encoding genes can be used for accurate population screening and surveillance of ESBL genes in symptomatic and asymptomatic infections. The applicability of the currently developed methodology is not limited to the detection of ESBL-encoding genes in the gut microbiome of *H*. *pylori*-infected patients, but can also potentially be applied to different samples, populations and various infection cases facing increased resistance to cephalosporins, amoxicillin, penicillin and other. In addition, these results suggest BL recolonization during restoration of the gut microbiome, implying that greater microbiome control would be necessary after antibiotic treatment. In conclusion, we believe that the ESBL screening panel is suitable for screening changes in the prevalence of ESBL coding genes, and in-depth research of the resistome is required to better understand the AMR gene reservoir in relation to antibacterial therapy, which could aid clinicians in choosing antibacterial therapy in the future.

## Supporting information

S1 File Appendix of statistical data. (DOCX)

S1 Table Primer sequences with the respective targeted ESBL clusters. (XLSX)

S2 Table Cluster IDs and NCBI derived annotations. (XLSX)

S3 Table Antimicrobial resistance genes, gene families and resistance mechanisms of the metagenomic sample set as detected by RGI and CARD reference database. (XLSX)
