# Supplementary material for: Abundance and prevalence of ESBL coding genes in patients undergoing first line eradication therapy for Helicobacter pylori
Source: PLoS One. 2023 Aug 10;18(8):e0289879. doi: 10.1371/journal.pone.0289879 (PMC10414638; doi:10.1371/journal.pone.0289879)
Supplement: S1 File — (DOCX) [file pone.0289879.s001.docx]

**Supplementary file 1**

**Development and use of an ESBL coding gene panel in patients undergoing first-line eradication therapy for *Helicobacter pylori***

**Authors**: Dita Gudra^1^, Ivars Silamikelis^1^, Janis Pjalkovskis^1^, Ilva Danenberga^1^, Darta Pupola^2^, Girts Skenders^2^, Maija Ustinova^1^, Kaspars Megnis^1^, Marcis Leja^2,3^, Reinis Vangravs^2^, Davids Fridmanis^1^.

1 – Latvian Biomedical Research and Study Centre, Ratsupites 1K-1, LV-1067, Riga, Latvia.

2 – Institute of Clinical and Preventive Medicine, University of Latvia, LV-1586, Riga, Latvia.

3 – Faculty of Medicine, University of Latvia, LV-1586, Riga, Latvia.

Correspondence and requests for materials should be addressed to:

Davids Fridmanis, e-mail: [davids@biomed.lu.lv](mailto:davids@biomed.lu.lv)


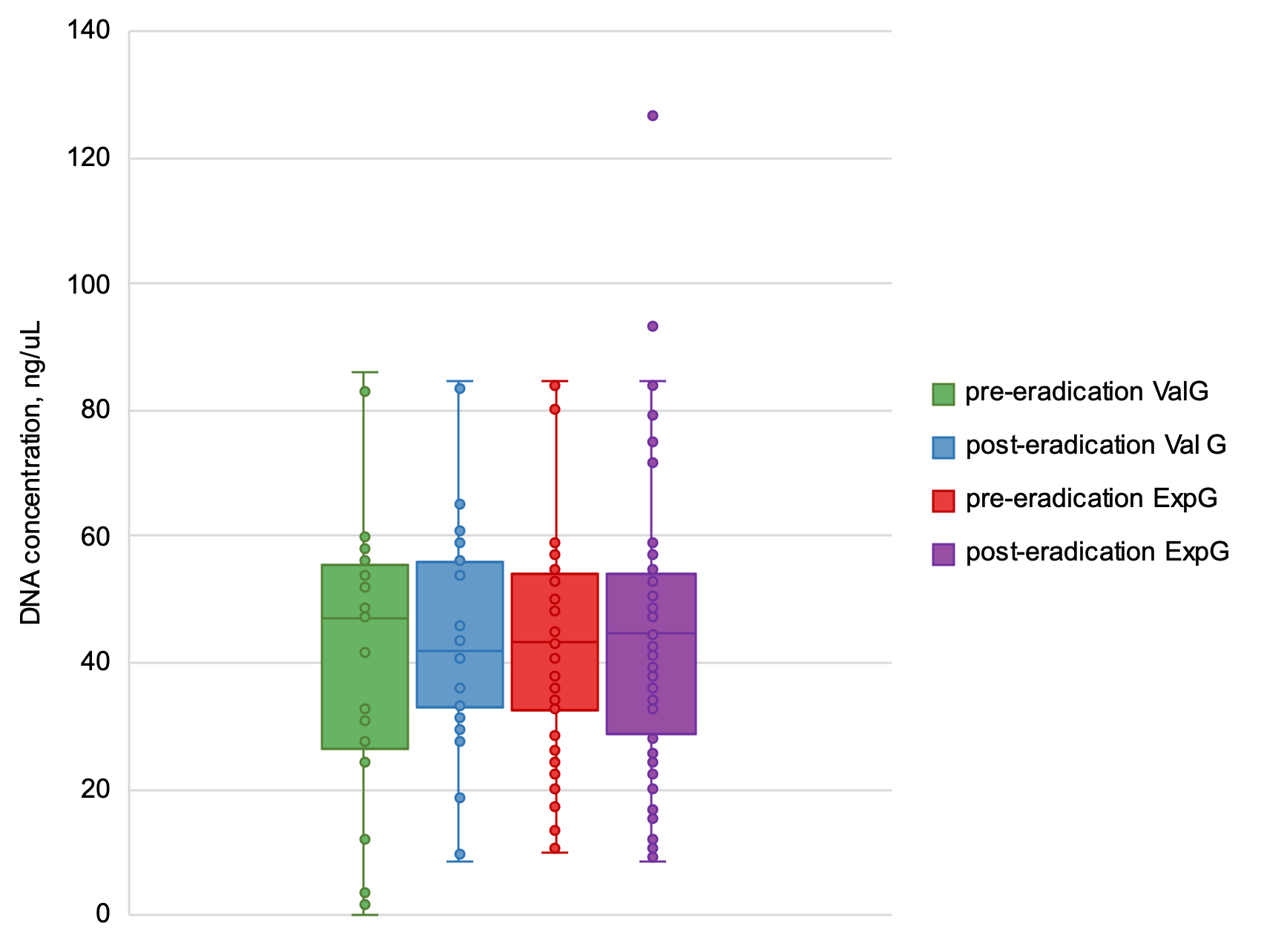


**Supplementary 1.1.** Mean DNA concentration per study group. Faecal samples were first lyophilised overnight, and microbial DNA was extracted using FastDNA SPIN Kit for Soil (MP Biomedicals, USA). Acquired quality and quantity of the extracted DNA was assessed using electrophoresis in 1.2% agarose gels and Qubit 2.0 Fluorometer (Life Technologies, USA). Abbreviations: pre-eradication ValG – gut microbiome samples collected in the pre-eradication state for the validation group; post-eradication ValG – samples collected in the post-eradication state for the validation group; pre-eradication ExpG – samples collected in the pre-eradication state for the experimental group; and post-eradication ExpG – samples collected in the post-eradication state for the experimental group.

**Supplementary 1.2.** Taxonomical summary of all samples at the genus level (bacteria, viruses, and eukaryotes).

**Supplementary 1.3.** Taxonomical summary of all samples at the bacterial species level.

**Supplementary 1.4.** Top 30 relative abundances of bacterial species by eradication state of the validation group. Each column represents bacterial species averaged from all individuals.

**Supplementary 1.5.** Estimated diversity characteristics of the validation group shotgun metagenomic analysis. Panel A – estimated alpha diversity metrics coloured by the treatment status and divided by gender. Panel B – non-metric multidimensional scaling of all samples, coloured by the treatment state and divided by gender. Abbreviations: F-post-erad - subjects in the post-eradication group with ineffective *H. pylori* eradication (HPE); F-pre-erad - subjects in pre-eradication group with ineffective HPE; S-post-erad - subjects in post-eradication group with successful HPE and S-pre-erad - subjects in pre-eradication group with successful HPE.

| **Shannon index** | |  |  |  |
| --- | --- | --- | --- | --- |
|  | F-post-erad | F-pre-erad | S-post-erad | All-post-erad |
| F-pre-erad | 1 | - | - | - |
| S-post-erad | 1 | 1 | - |  |
| S-pre-erad | 1 | 0.736 | 0.019 |  |
| All-pre-erad | - | | | 0.019 |
|  |  |  |  |  |
| **Chao1 index** | |  |  |  |
|  | F-post-erad | F-pre-erad | S-post-erad | All-post-erad |
| F-pre-erad | 1 | - | - | - |
| S-post-erad | 1 | 1 | - |  |
| S-pre-erad | 1 | 1 | 0.85 |  |
| All-pre-erad | - | | | 0.21 |
|  |  |  |  |  |
| **Observed OTUs** | |  |  |  |
|  | F-post-erad | F-pre-erad | S-post-erad | All-post-erad |
| F-pre-erad | 1 | - | - | - |
| S-post-erad | 1 | 1 | - |  |
| S-pre-erad | 1 | 1 | 1 |  |
| All-pre-erad | - | | | 0.29 |

**Supplementary 1.6.** Pairwise comparisons using Wilcoxon rank sum test and holm P-value adjustment method for alpha diversity metrics. Abbreviations: F-post-erad - subjects in the post-eradication group with ineffective *H. pylori* eradication (HPE); F-pre-erad - subjects in pre-eradication group with ineffective HPE; S-post-erad - subjects in post-eradication group with successful HPE; S-pre-erad - subjects in pre-eradication group with successful HPE; All-pre-erad – all pre-eradication group samples; All-post-erad – all post-eradication group samples.

**Supplementary 1.7.** Significantly associated gene ontology (GO) entries from metagenomic data with the treatment state of *H.pylori* positive patients before and after eradication therapy. Decipherments of GO IDs: GO:0006811 – ion transport; GO:0035442 – dipeptide transmembrane transport; GO:0034219 – carbohydrate transmembrane transport; GO:0009307 – DNA restriction-modification system; GO:0042274 – ribosomal small subunit biogenesis; GO:0042777 – plasma membrane ATP synthesis coupled proton transport; GO:0015693 – magnesium ion transport; GO:0001123 – DNA-templated transcription, initiation; GO:0019242 – methylglyoxal biosynthetic process; GO:0009236 – cobalamin biosynthetic process; GO:0019243 – methylglyoxal catabolic process to D-lactate via S-lactoyl-glutathione; GO:0006817 – phosphate ion transport; GO:0045226 – extracellular polysaccharide biosynthetic process; GO:0030643 – cellular phosphate ion homeostasis; GO:0019568 – arabinose catabolic process; GO:0006168 – adenine salvage; GO:0006231 – dTMP biosynthetic process; GO:0006541 – glutamine metabolic process.
